# The effect of game-based interventions on children and adolescents with autism spectrum disorder: A systematic review and meta-analysis

**DOI:** 10.3389/fped.2025.1498563

**Published:** 2025-04-04

**Authors:** Jiaxin Gao, Wei Song, Dunbing Huang, Anren Zhang, Xiaohua Ke

**Affiliations:** ^1^ School of Health and Rehabilitation, Chengdu University of Traditional Chinese Medicine, Chengdu, China; ^2^ Department of Rehabilitation Medicine, Shanghai Fourth People’s Hospital Affiliated to Tongji University, Shanghai, China

**Keywords:** autism spectrum disorder, game-based interventions, children, adolescence, meta-analysis

## Abstract

**Purpose:**

The purpose of this meta-analysis was to conduct a comprehensive evaluation of randomized controlled trials (RCTs) of game-based interventions (GBI) for children and adolescents with autism spectrum disorder (ASD) to identify the clinical efficacy of GBI on core symptoms and other concomitant symptoms of ASD.

**Methods:**

PubMed, Web of Science, Embase and the Cochrane Library were systematically searched for articles published until July 2023.

**Results:**

Twenty-four studies with 1,801 patients met the inclusion criteria. The results showed that GBI had a significant positive effect on social skills (*g* = −0.59, *p* = 0.004), social behaviors (*g* = 0.45, *p* < 0.001), and cognition (*g* = 0.57, *p* < 0.001) in children and adolescents with ASD, while the effects of language expression (*g* = 0.15), anxiety (*g* = −0.13), and parenting stress (*g* = −0.51) were small and nonsignificant.

**Conclusions:**

The results of the current meta-analysis showed that GBI was effective in improving social skills, social behaviors and cognition in children and adolescents with ASD in the existing studies and was not significant in improving language skills, anxiety and parental stress, but due to the limited number and low quality of the included studies, the above conclusions need to be validated by conducting more large-sample, high-quality RCTs.

**Systematic Review Registration:**

www.crd.york.ac.uk/prospero/display_record.php?ID=CRD42023390793, identifier: CRD42023390793.

## 1 Introduction

Autism spectrum disorder (ASD) is a neurodevelopmental disorder that is characterized by social deficits and repetitive stereotyped behaviors, and most children with ASD have difficulty acquiring language skills and communicative gestures (1, 2). However, recent studies have gradually revealed that individuals with autism not only face these challenges but also exhibit numerous unique strengths. For example, they often demonstrate strong memory abilities, keen observational skills, a heightened focus on specific areas of interest, and exceptional performance in logical reasoning and systematic thinking (3–5). A 2012 review commissioned by the World Health Organization estimated the global prevalence of ASD to be approximately 1% (6), and a 2017 study reported prevalence rates in developed countries to be at least 1.5% (7). The prevalence of ASD has shown an increasing trend from year to year (8). ASD is often accompanied by physical, mental, neurodevelopmental, and functional conditions, such as attention deficit hyperactivity disorder, intellectual disability, emotional disorders, speech and language delays, learning difficulties, and different forms of anxiety (9–11), with significant and negative impacts on the well-being of the child or adolescent and his or her family (12), which is a cause for widespread concern in the whole community. In addition, some children with ASD have social disorders that may persist into adolescence or even adulthood, affecting their schooling and employment (7, 13). Therefore, early intervention and treatment for children and adolescents with ASD is critical.

ASD, as a complex neurodevelopmental disorder, requires a comprehensive, multidisciplinary intervention strategy to meet the unique needs of this population. These strategies should not only focus on symptom reduction but also fully leverage the strengths of individuals with ASD to promote their overall development. Treatment strategies include behavioral interventions, speech and language therapy, occupational therapy, social skills training, and game-based Interventions (GBI) (14–16). Behavioral interventions, represented by applied behavior analysis, shape target behaviors through reinforcement mechanisms and reduce maladaptive behaviors (14, 17). Speech and language therapy focuses on enhancing verbal and non-verbal communication (18, 19), techniques may include using augmentative and alternative communication systems, such as picture exchange communication system, especially for non-verbal children (16). Occupational therapy enhances children's independence in daily activities through sensory integration training and fine motor skills training, improving sensory processing and motor coordination (20, 21). Social skills training helps children understand social rules, improve social interaction abilities, and reduce feelings of loneliness through group activities or one-on-one guidance (22–24). GBI have gained increasing attention in recent years as a person-centered approach (25). This intervention encourages active participation through play activities, not only improving core symptoms but also leveraging the strengths of individuals with ASD through its flexibility and fun, making it increasingly valued in ASD treatment.

GBI are systematic interventions implemented through play mediums, aimed at improving the social, communication, emotional regulation, cognitive development, and behavioral management skills of individuals with ASD (25, 26). These interventions leverage the strengths of individuals with autism, combining interest-driven and task-oriented approaches to provide a safe and motivating environment that fosters holistic development (27, 28). For example, many individuals with autism excel in rule-based activities and logical reasoning, and structured play, such as board games or puzzles, effectively stimulates their systematic thinking abilities while enhancing social interactions and emotional expression (29–31). Free play provides children with space for self-directed choices, encouraging spontaneity and creativity. This unstructured form offers opportunities for observation and guidance, helping to enhance an individual's initiative and social skills (32, 33). Digital game-based interventions, such as virtual reality (VR) and gamified platforms, use multisensory experiences, including visual and auditory stimuli, to assist children in learning emotional recognition and social interaction skills in areas of interest (34, 35). Furthermore, therapeutic play, designed and implemented by professional therapists according to individual needs, provides in-depth interventions targeting specific developmental areas, these games may utilize an individual's sensitivity to details to support progress in emotional regulation and task completion (36, 37). Compared to other forms of intervention, GBI also provide immediate feedback and reinforcement mechanisms, which can help individuals with autism build confidence and a sense of self-efficacy (38). In the game, their strengths can be fully recognized and utilized, further motivating their performance in other life situations (39). In this field, play therapy, as a subfield of GBI, demonstrates higher levels of professionalism and standardization. According to the definition by the Association for Play Therapy, play therapy is provided by professionally certified therapists, based on specific theoretical frameworks, and utilizes play as a medium to help individuals prevent or resolve socio-psychological difficulties while promoting their optimal growth and development (40). The forms of GBI intervention include one-on-one GBI, led individually by a therapist, focusing on specific goals for the child, such as language development or social skills (26, 41); group games involving multiple children, with the goal of promoting social skills through cooperative play (42, 43); gamified digital technologies, such as VR games and interactive applications, which enhance the intervention's fun and technological appeal while providing real-time feedback; and natural environment-based play, where interventions are conducted outdoors or in family settings, helping children transfer skills to real-life situations (41).

A large amount of research has reported significant improvements in social skills, social behaviors, cognition, language functioning, and emotion in children and adolescents with ASD who undergo GBI (30, 44–46), initially suggesting a positive effect of GBI on children and adolescents with ASD. However, the strength of the evidence is insufficient due to limitations in sample sizes as well as trial designs. There is no high-level meta-analysis evidence to validate the effectiveness of GBI for ASD; therefore, the purpose of this study was to provide a comprehensive overview and evaluation of randomized controlled trials (RCTs) of GBI for children and adolescents with ASD and to conduct a meta-analysis of outcomes in the domains of social, cognition, language, emotion and parenting stress.

## 2 Method

Our protocol was registered on the International Prospective Register of Systematic Reviews (PROSPERO) and assigned registration number CRD42023390793. To take the highest quality approach, we followed the Cochrane Handbook guidelines (Version 6.2) for conducting “Overview of Reviews” and the PRISMA statement in reporting our meta-analysis.

### 2.1 Criteria for selecting meta-analyses

#### 2.1.1 Types of studies

Studies eligible for inclusion were RCTs published in English.

#### 2.1.2 Types of participants

Participants were children and adolescents who had been definitively diagnosed with ASD by a medical professional or school psychologist using a recognized diagnostic procedure, did not have any intellectual disability and were younger than 18 years of age; those younger than 10 years of age were considered children, and those 10–18 years of age were considered adolescents (47, 48).

#### 2.1.3 Types of interventions

The treatment group received a game-based intervention, which included the following categories: structured play, games with specific goals and rules, such as role-playing, puzzles, etc.; free play, where children could freely choose their play activities; digital GBI, such as gamified digital platforms or virtual reality technology; and therapeutic play, designed and implemented by professional therapists for in-depth intervention in specific developmental areas. The control group received therapies other than play or was placed on a waitlist control.

#### 2.1.4 Types of outcome measures

Our primary outcome measure was the social skills score, and secondary outcome measures included the AODS scale, social behaviors, cognition, language understanding and expression, anxiety, and parenting stress. When more than one continuous measure was described for the same outcome in the same study, the primary outcome was used. For studies that met the inclusion criteria, postintervention means, standard deviations, and sample sizes of participants who completed the studies were extracted.

### 2.2 Search strategy

We conducted a systematic literature search in PubMed, Web of Science, Embase and the Cochrane Library for all published studies up to July 2023 on the effects of GBI on children and adolescents with ASD. The following terms were used as text words and key words: (Autism Spectrum Disorder OR Autism Spectrum Disorders OR Autistic Spectrum Disorder OR Autistic Spectrum Disorders) AND (children OR Child OR adolescent OR Adolescents OR Adolescence OR Teens OR Teen OR Teenagers OR Teenager OR Youth OR Youths) AND (play therapy OR game therapy OR Play Therapies OR Therapies, Play OR Therapy, Play OR Play-based Mental Health Intervention OR Play based Mental Health Intervention OR Sandplay Therapy OR Sandplay Therapies OR Therapies, Sandplay OR Therapy, Sandplay OR Sandplay).

### 2.3 Data extraction

All retrieved literature was first imported into EndNote X9, and duplicates were removed using this software. Four evaluators (JXG, WS) then independently conducted literature screening, data extraction, and exclusion of literature that did not meet the inclusion criteria, and the results were cross-checked between evaluators. Study characteristics and outcome measures were extracted by JXG and checked by WS. The following data were extracted into a table: author, year of publication, country, sample size, age range, interventions in the treatment and control groups, intervention duration, evaluation measures and intervention outcomes. If data relevant for our analyses were missing in the included meta-analyses, we attempted to contact the corresponding author of the published meta-analysis up to three times to seek the required data from the author. If data remained unavailable, that meta-analysis was excluded.

### 2.4 Quality of included meta-analyses

Quality and risk of bias were assessed using the Cochrane Collaboration “Risk of Bias” tool (49), including (i) random sequence generation; (ii) allocation concealment; (iii) blinding of participants and personnel; (iv) blinding of outcome assessment; (v) incomplete outcome data; (vi) selective reporting; and (vii) other bias.

Two evaluators (JXG and WS) individually assessed the quality of the selected studies, including “low-risk”, “high-risk” and “unclear” judgments, with cross-checking at the end of the process, and the divergent literature was reviewed with the assistance of XHK. The risk of bias was assessed in RevMan 5.4 software, and the summary of risk bias in the included studies was plotted as well as the single study bias risk analysis.

### 2.5 Meta-meta-analytic procedure, data synthesis, and statistical analysis

#### 2.5.1 Meta-meta-analytic procedure

The meta-analytic procedures used in this paper adhered to all applicable Preferred Reporting Items for Systematic Reviews and Meta-Analyses guidelines for meta-analysis (50).

#### 2.5.2 Calculation of effect sizes

Comprehensive meta-analysis (version 3.0, Biostat Inc.) was used to calculate individual study and pooled effect sizes. The effect sizes were represented using Hedges' *g*, which was calculated by dividing the difference between the means of the treatment and control groups by the standard deviation and weighted according to sample size to correct for small sample bias (51). The 95% confidence intervals for the effect sizes were reported. Effect sizes of 0.2, 0.5, and greater than 0.8 were considered small, medium, and large effects, respectively (52). A random-effects model was used in all calculations.

#### 2.5.3 Testing homogeneity

The Q test informs the presence vs. the absence of heterogeneity, and the I^2^ index is proposed to quantify the degree of heterogeneity. The Q test indicates the presence of heterogeneity by summing the squared deviation of each study's effect size from the overall effect size and weighing each study by its variance, the *I*^2^ quantifies the degree of heterogeneity by providing the percentage of the amount of variance attributable to between-study variability (53). A value of 0% indicates no heterogeneity, while 25%, 50% and 75% indicate low, moderate, and high heterogeneity, respectively. If heterogeneity was high, we conducted sensitivity analyses using leave-one-out analysis, and we assessed the influence on the remaining heterogeneity of overall effects. After identifying outliers, we calculated the effect size again with those outliers removed.

#### 2.5.4 Subgroup analysis

In the meta-analysis of primary outcome indicators, subgroup analysis was performed by age of the participants, and meta-analysis was performed for the two subgroups of children and adolescents in social skills scores. In the meta-analysis of cognition, subgroup analysis was performed by the cognitive type of the participants, which was categorized into the two subgroups of attention and facial memory.

#### 2.5.5 Testing for and managing publication bias

We detected publication bias by funnel plots (54) and Egger regression tests (55). If the funnel plot visually shows some asymmetry and Egger's regression test results are significant, this indicates publication bias (56). In cases of publication bias, we utilized a “trim and fill” procedure to derive adjusted effect size estimates. This procedure provided the best estimates of unbiased effect sizes to address potential publication bias and selective outcome reporting bias. If the effect is no longer significant, then publication bias is suspected (57).

## 3 Result

### 3.1 Study selection

**Figure 1:**
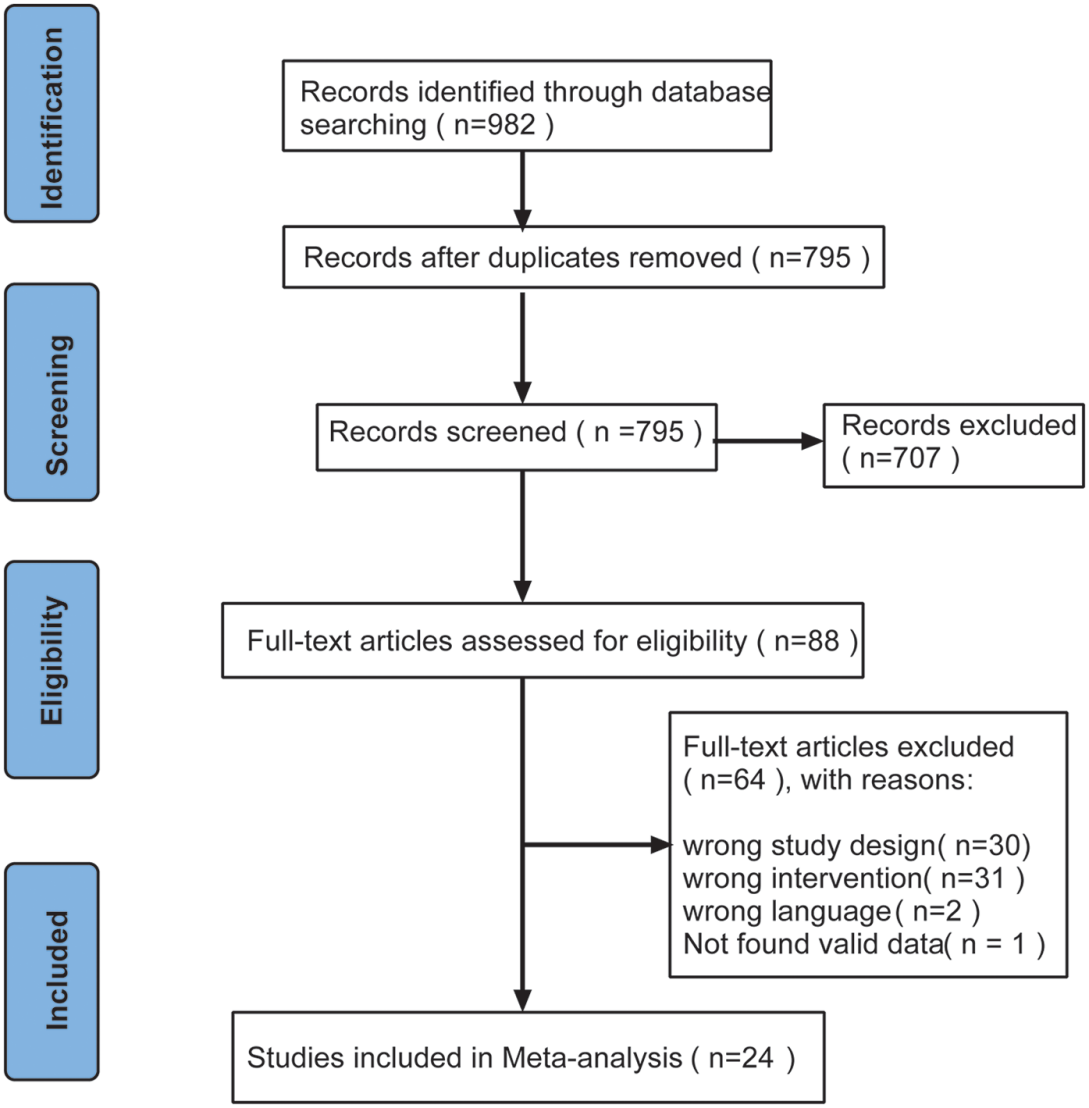
Flow chart of the literature search and screening process.

A total of 982 studies were retrieved from the database, duplicates (*n* = 187) were excluded, and the remaining (*n* = 795) studies were screened by reading the abstracts. After screening, 707 studies did not meet the inclusion criteria for this meta-analysis and were therefore excluded. Subsequently, the full text of the remaining 88 studies was screened, of which 64 studies did not meet the inclusion criteria and were excluded. Finally, 24 studies were included in the meta-analysis. The specific flow chart is shown in Figure 1.

### 3.2 Descriptive characteristics

The main characteristics of the included studies are summarized in Table 1. For the 24 studies included in the overall meta-analysis, publication years were from 2006–2023. Of these, 12 were from the United States, 4 from China, 3 from the United Kingdom, 2 from Australia, and 1 each from the Netherlands, Canada, and Serbia. All data were collected from 1,801 participants. The sample size ranged from 10–290 participants, the age range of the participants was 2.8–18 years old, and the duration of the intervention ranged from 4 weeks–12 months. The interventions in the treatment group were all game-based (e.g., video games, symbolic games, role-playing, sandplay therapy), and the interventions in the control group were either interventions other than game therapy or wait-list control. For outcome measures, 9 studies evaluated social skills using SRS scales (25, 26, 58–64), 2 studies reported ADOS scores (65, 66), 10 studies evaluated social behaviors (25, 61, 62, 65, 67–72), 6 studies evaluated cognitive level (27, 61, 63, 70, 73, 74), 3 and 5 studies evaluated language understanding (65, 70, 74) and language expression (63, 65, 68, 70, 74), respectively, and 3 studies each evaluated emotions (67, 75, 76) and parenting stress (60, 70, 74). Only 1 study in the final outcome reported no visible impact, and the remaining studies reported positive effects of GBI on social, emotional, language or cognition in children and adolescents with ASD.

**Table 1:** Characteristics of included studies.

No.	Author	Year	Country	Number	Age	Treatment group	Control group	Duration	Mesure	Outcome
1	Yordan Penev, et al. (62)	2021	United States	72	3–12Y	Mobile game	Other procedures	4W	SRS-2, VABS-II	Improve socialization
2	Connie Kasari, et al. (27)	2006	United States	58	3–4Y	Symbolic play	Control condition	5–6W	ESCS, SPA, Mother–child interactions	More diverse types of symbolic play in interaction with their mothers and higher play levels on both the play assessment and in interaction with their mothers.
3	Gerald Mahoney, et al. (66)	2016	United States	112	2Y8M–5Y11M	Play session	Community standard treatment	12M	ADOS-CSS	Improve social engagement
4	Renae Beaumont, et al. (67)	2021	Australia	70	7Y–12Y	Video game	Cognitive skills training	10W	SSQ-P, ERSSQ-P, SCAS-P, ECBI	Improve social and emotion skills
5	Jason W. Griffin, et al. (25)	2021	United States	40	10–18Y	Serious game	Standard care	10W	Gaze Perception task, SSIS, SRS-2	Improve eye gaze cues and social skills
6	Lieke A M W Wijnhoven, et al. (76)	2020	Netherlands	109	8–16Y	Video game	Triple Town	6W	SCAS-C, SCAS-P, ADIS-P	Decrease in anxiety symptoms
7	Sanja Vukićević, et al. (77)	2019	Serbia	10	9–13Y	visuo-motor games	Standard treatment	5W	PDMS-2, GARS-3, DASH-2	Improvement in gross motor skills and successful generalization of acquired skills
8	Richard Solomon, et al. (70)	2014	United States	128	2Y8M–5Y11M	Play session	Usual community services	12M	ADOS-G, PSI, FEAS, SCQ–MCDI	Made greater improvements in their interaction, functional development, and autism symptomatology
9	Guihua Liu, et al. (60)	2023	China	52	3–6Y	Parent-Child Sandplay	Applied Behavior Analysis-based program	20W	ABC, SRS, PSI-SF, CSHQ	Social development
10	Blythe A Corbett, et al. (75)	2017	United States	30	8–14Y	Role-playing	Wait-list control	10W	STAI-C	Improvement in social competence in youth with ASD and reduction in trait-anxiety associated with more social interaction with peers
11	Blythe A Corbett, et al. (68)	2019	United States	77	8–16Y	Role-playing	Wait-list control	10W	TOM, IFM, ERP	Improvement in social cognition and behavior
12	Blythe A Corbett, et al. (63)	2023	United States	290	10–16Y	Role-playing	Tackling Teenage Training	10W	IFM, CASS, SRS-2	Increase social interest and key social behaviors
13	Blythe A Corbett, et al. (61)	2016	United States	30	8–14Y	Role-playing	Wait-list control	10W	SRS, ABAS, MFD, TOM	Improvement in core areas of social competence
14	Ellen A. Doernberg, et al. (73)	2021	United States	25	6–9Y	Pretend play	Wait-list control	5W	APS, KAI-R	Significantly increased in imagination and cognitive play skills
15	Sue Fletcher-Watson, et al. (65)	2016	United Kingdom	49	under 6Y	Video game	Wait-list control	2M	ADOS-2, BOSCC, MCDI, CSBS-DP	Did not have an observable impact on real-world social communication skills
16	Connie Kasari, et al. (74)	2015	United States	86	22–36M	Symbolic play	PEI	10W	Time Joint Engaged, Initiating Joint Attention, MSEL, RDLS, PSI	Improvements in functional-play diversity, overall play level and language skills
17	Cally Kent, et al. (69)	2021	Australia	68	6–11Y	Video-modeling game	Wait-list control	10W	ToP, Piers-Harris 2, HCSBS, PRQ, SSBS	Improve play
18	Guo-Kai Li, et al. (58)	2019	China	44	5–6Y	Integrated sandplay therapy	Regular training	6M	SRS, ERA	Improve social responsiveness and emotion recognition ability
19	Gui-Hua Liu, et al. (59)	2019	China	50	3–6Y	Integrated sandplay therapy	Structured teaching and auditory integration training	2M	ABC, CARS, SRS, CSHQ, ERA, PSI-SF	Improve the core symptoms and sleep quality
20	Sarah J Macoun, et al. (79)	2021	Canada	20	6–12Y	Serious game	Wait-list control	8W	TAP	Improvement in attention, executive function, emotion-regulation, flexibility, communication, and social skills.
21	April A. Schottelkorb, et al. (26)	2020	United States	23	4–10Y	Child-centered play therapy	Wait-list control	6W	SRS-2, CBCL	Decrease in ASD core symptoms and behavioral symptoms
22	Wing-Chee So, et al. (64)	2020	China	23	4–6Y	Robot-based play-drama	Wait-list control	9W	ESCS, SPA, SRS	Improvements in joint attention initiations and functional play behaviors
23	Han-I Wang, et al. (71)	2022	United Kingdom	248	7–15Y	LEGO-based therapy	Usual support	52W	EQ-5D-Y, CHU-9D	Reduction in costs and improvement in quality-adjusted life years
24	Barry Wright, et al. (72)	2023	United Kingdom	217	7–15Y	LEGO® play	Usual support	7–15W	SSIS	Positive effect on social and emotional skills

**Note.** ABC, autism behavior checklist; ABAS, adaptive behavior scales; ADIS-P, anxiety disorders interview schedule-parent; ADOS-CSS, autism diagnostic observation schedule-calibrated severity scores; ADOS-G, autism diagnostic observation schedule-generic; APS, affect in play scale; ABC, autism behavior checklist; BOSCC, brief observation of social communication change; CARS, children autism rating scale; CASS, contextual assessment of social skills; CBCL, child behavior checklist; CSHQ, children's sleep habits questionnaire; CSBS-DP, communication and symbolic behaviour scale-developmental profile; DASH-2, developmental assessment for individuals with severe disabilities; ECBS, functional emotional assessment scale; ERSSQ, emotion regulation and social skills questionnaire; ESCS, early social-communication scales; FEAS, functional emotional assessment scale; GARS-3, Gilliam autism rating scale; IFM, incidental face memory; MCDI, MacArthur communicative development inventory; MCDI, MacArthur communicative development inventory; MFD, memory for faces delayed; MSEL, Mullen scales of early learning; PDMS-2, Peabody developmental motor scales; PEI, parent-only psychoeducational intervention; PRQ, parenting relationship questionnaire; PSI, parenting stress index; RDLS, Reynell developmental language scales; SCAS-C, Spence children's anxiety scale—children; SCAS-P, Spence children's anxiety scale—parent; SCQ, social communication questionnaire; SRS, social responsiveness score; SSBS, school social behavior scales; SSIS, social skills improvement system; SSQ, social skills questionnaire; SPA, structured play assessment; TAP, test of attentional performance; TOM, theory of mind; ToP, test of playfulness; VABS-II, vineland adaptive behavior scales-II; W, week; Y, year.

### 3.3 Risk of bias

**Figure 2:**
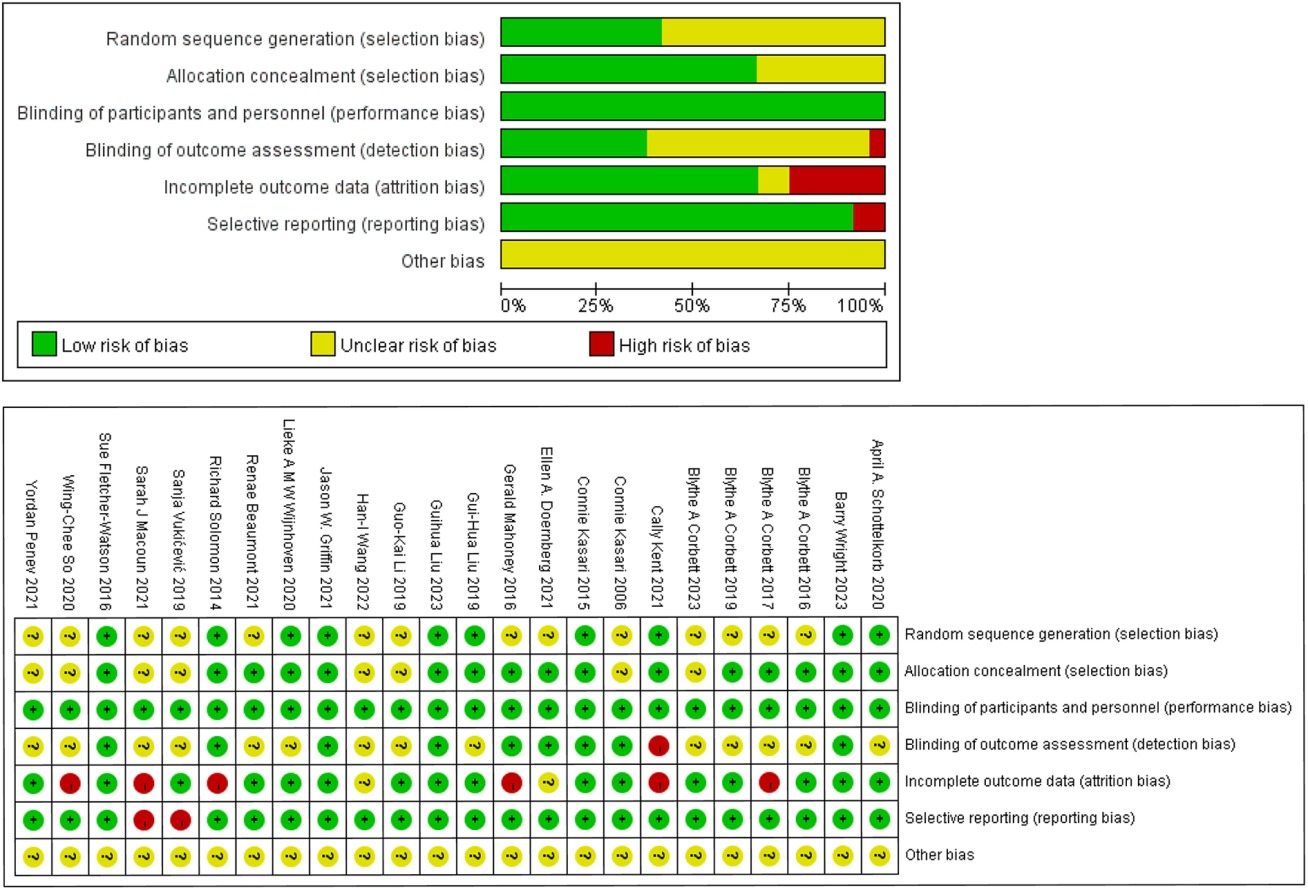
Risk of bias graph.

The quality and risk of bias of each study was assessed using the Cochrane Collaboration “Risk of bias” tool. Ten studies reported randomization and explicitly reported methods of randomization (e.g., random number table method, random number generator, etc.) that ensured comparability between groups. The remaining study 1 (62) did not have enough details to assess how groups were randomized, and 13 did not explicitly mention randomization methods. Similarly, 16 studies (67%) demonstrated appropriate concealment of allocation, suggesting that 33% of studies did not report enough information to determine whether subjects and researchers could have predicted the outcome of the intervention's allocation. None of the studies were blinded to the subjects but were judged to be “low risk” because it was unlikely that the outcome would be affected by the absence of blinding. Nine studies reported that outcome assessment was conducted by a completely uninformed third party, 14 studies did not mention whether outcome assessment was blinded, and in 1 study, both the outcome assessment and the intervention were conducted by the same therapist, which was a process judged to be “high risk”. Six studies (25%) with a high risk of incomplete outcome data did not specify the reason for their missingness, and the remaining 18 studies (75%) were either not missing or the reason for their missingness was clear and unlikely to be associated with the true value of the outcome. Selective reporting had a low risk of bias in 22 studies (92%), and 2 studies that did not report all of the prespecified outcome indicators were judged to be high risk. There was insufficient information to evaluate whether there were other significant risks of bias, so “other bias” was judged to be unclear. The risk of bias graph in the included studies and the risk of bias summary in the included studies are shown in Figure 2.

### 3.4 Meta-analysis

The current study included six independent meta-analyses, including the primary outcome indicator of social skills score, as well as other secondary measures such as ADOS scores, social behaviors, cognition, language understanding, language expression, anxiety and parenting stress. Table 2 provides a summary of each outcome's results.

**Table 2:** Summary of each outcome's results.

Informant	k	Total N	Effect size(*g*)	*p*-Value	Q	*I*-squared
Social skills	9	542	−0.59	0.004**	36.09	77.84
Children	6	313	−0.81	0.001**	16.63	69
Adolescents	3	229	−0.16	0.52	4.91	59
ADOS-SA	2	161	−0.11	0.68	2.73	63.42
ADOS-RRS	2	161	0.05	0.74	0.22	0.00
Social Behaviors	10	894	0.45	0.00**	14.46	37.77
Cognition	6	418	0.57	0.00**	4.91	0.00
Attention	3	174	0.66	0.00**	1.7	0.00
Memory	2	219	0.57	0.03*	1.83	45.47
Language Understanding	3	263	−0.02	0.89	0.85	0.00
Language Expression	5	439	0.15	0.15	4.42	9.58
Anxiety	3	203	−0.13	0.37	0.88	0.00
Partenting Stress	3	186	−0.51	0.18	8.3	75.9

N, Number.

* *p* < 0.05.

** *p* < 0.01.

#### 3.4.1 Social skills

**Figure 3:**
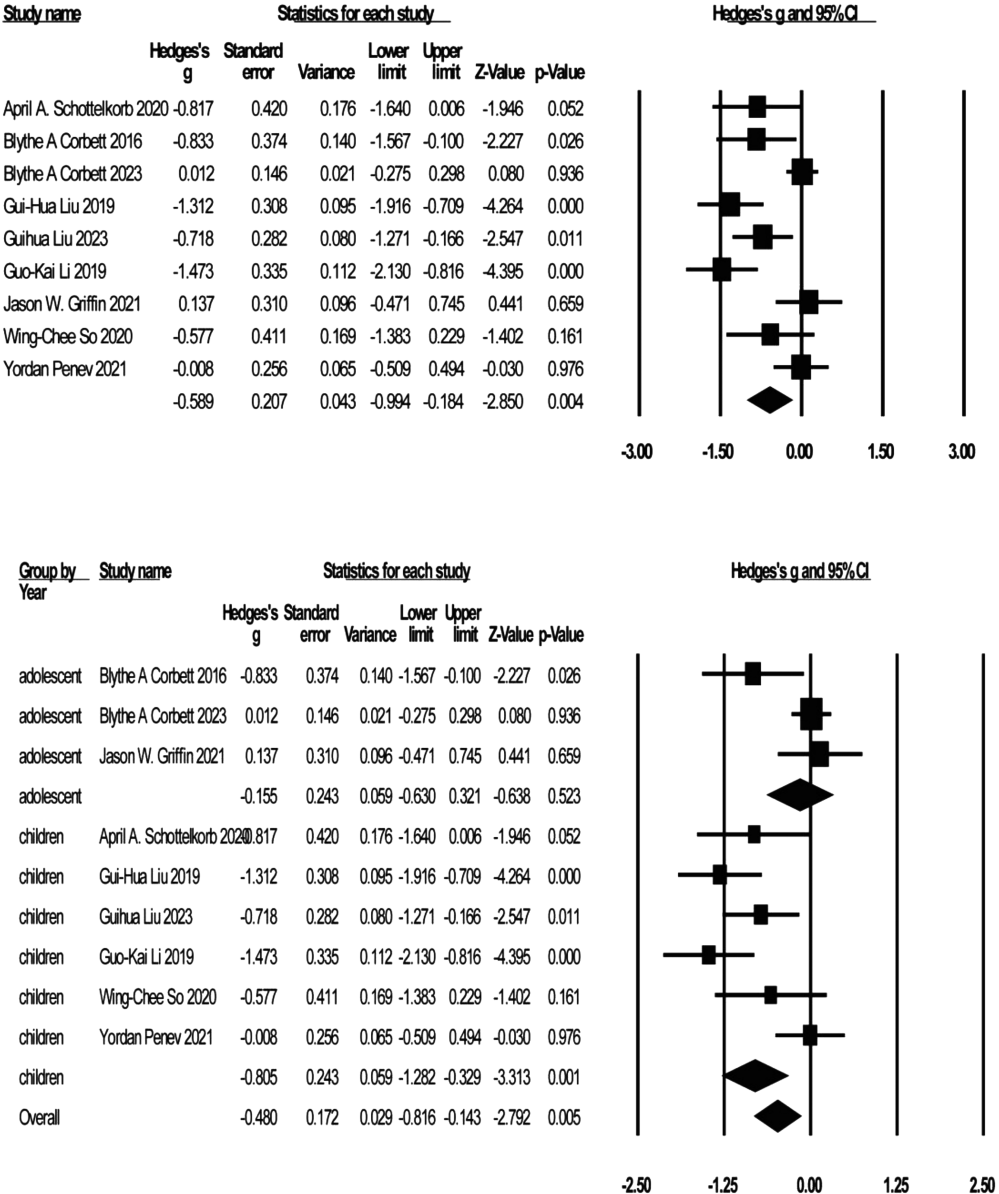
Forest plot of meta-analysis of social skills.

Nine studies reported on the social skills of children and adolescents with ASD by SRS scores (see Figure 3). The negative effect suggests that the improvement in social skills, and the effect sizes ranged from *g* = −1.47 to *g* = 0.14. Participants treated with GBI had significantly improved social skills scores compared to the control group [*g* = −0.59, K = 9, 95% CI (−0.99, −0,18), Z = −2.85, *p* = 0.004]. Moderate heterogeneity was noted across studies (*I*^2^ = 77.84). Sensitivity analyses identified three studies (25, 62, 63) that were considered outliers with the greatest impact on interstudy heterogeneity. Excluding three studies resulted in a significant reduction in heterogeneity (*I*^2^ = 8.33%). The effect sizes were again recombined [*g* = −0.98, 95% CI (−0.27, −0.70), Z = −6.68, *p* = 0.000]. The nine studies were categorized into child and adolescent groups according to age, and subgroup analyses were conducted by group, which showed significant improvement in social skill scores in the child group [*g* = −0.81, *K* = 6, 95% CI (−1.28, −0.33), *Z* = −3.31, *p* = 0.001, *I*^2^ = 69%] and no statistically significant improvement in social skills in the adolescent group [*g* = −0.16, *K* = 3, 95% CI (−0.63, 0.32), *Z* = −0.64, *p* = 0.52, *I*^2^ = 59%].

#### 3.4.2 ADOS

**Figure 4:**
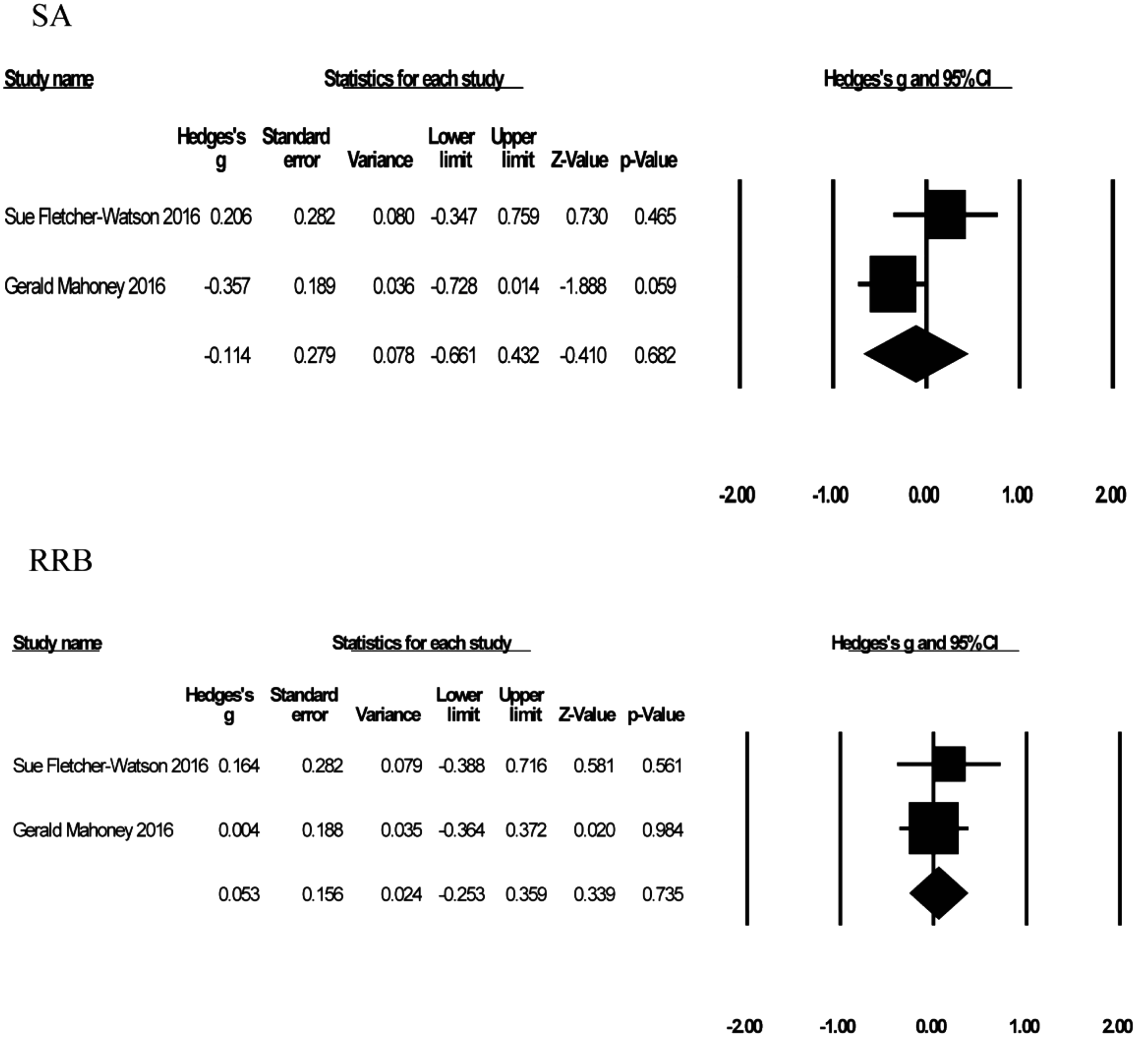
Forest plot of the meta-analysis of ADOS.

Two studies with 161 participants reported the effect of GBI on ADOS scores (see Figure 4), but because one study reported scores on only two domains of ADOS, social affect (SA) and restricted and repetitive behavior (RRB), and not total ADOS scores, we conducted independent meta-analyses of the ADOS SA and ADOS RRB domains. SA domain score effect sizes ranged from *g* = −0.21 to *g* = −0.36, and RRB domain score effect sizes ranged from *g* = 0.004 to *g* = 0.16, with negative effects representing improvements in ADOS scores. Compared with the control group, participants who received treatment had improved ADOS SA scores [*g* = −0.11, *K* = 2, 95% CI (−0.66, 0.43), *Z* = −0.41, *p* = 0.68] but not ADOS RRB scores [*g* = 0.05, *K* = 2, 95% CI (−0.25, 0.36), *Z* = 0.339, *p* = 0.74]. Heterogeneity was tested for ADOS SA (*I*^2^ = 63.42%) and ADOS RRB (*I*^2^ = 0%), and although mild to moderate heterogeneity existed for ADOS SA, the study sample was too small to allow sensitivity analysis.

#### 3.4.3 Social behaviors

**Figure 5:**
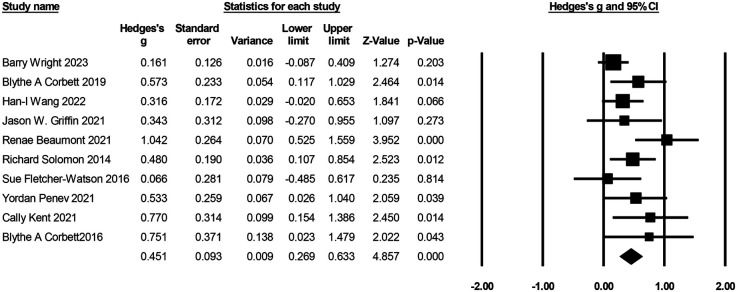
Forest plot of the meta-analysis of social behaviors.

Ten studies with 894 participants reported on the effects of GBI on social behaviors (see Figure 5). Effect sizes ranged from *g* = 0.07–*g* = 1.04, with positive effects indicating increased social behaviors. Compared to the control group, participants treated with GBI achieved significantly greater improvements in social behavior scores [*g* = 0.45, *K* = 10, 95% CI (0.27, 0.63), *Z* = 4.86, *p* = 0.00]. Heterogeneity test (*I*^2^ = 37.77%), mild to moderate heterogeneity. Sensitivity analyses identified 1 meta-analysis (67) as an outlier, and when the study was excluded, heterogeneity (*I*^2^ = 1.61) and reanalysis of the results were observed [*g* = 0.36, *K* = 9, 95% CI (0.22, 0.50), *Z* = 4.96, *p* = 0.00].

#### 3.4.4 Cognition

**Figure 6:**
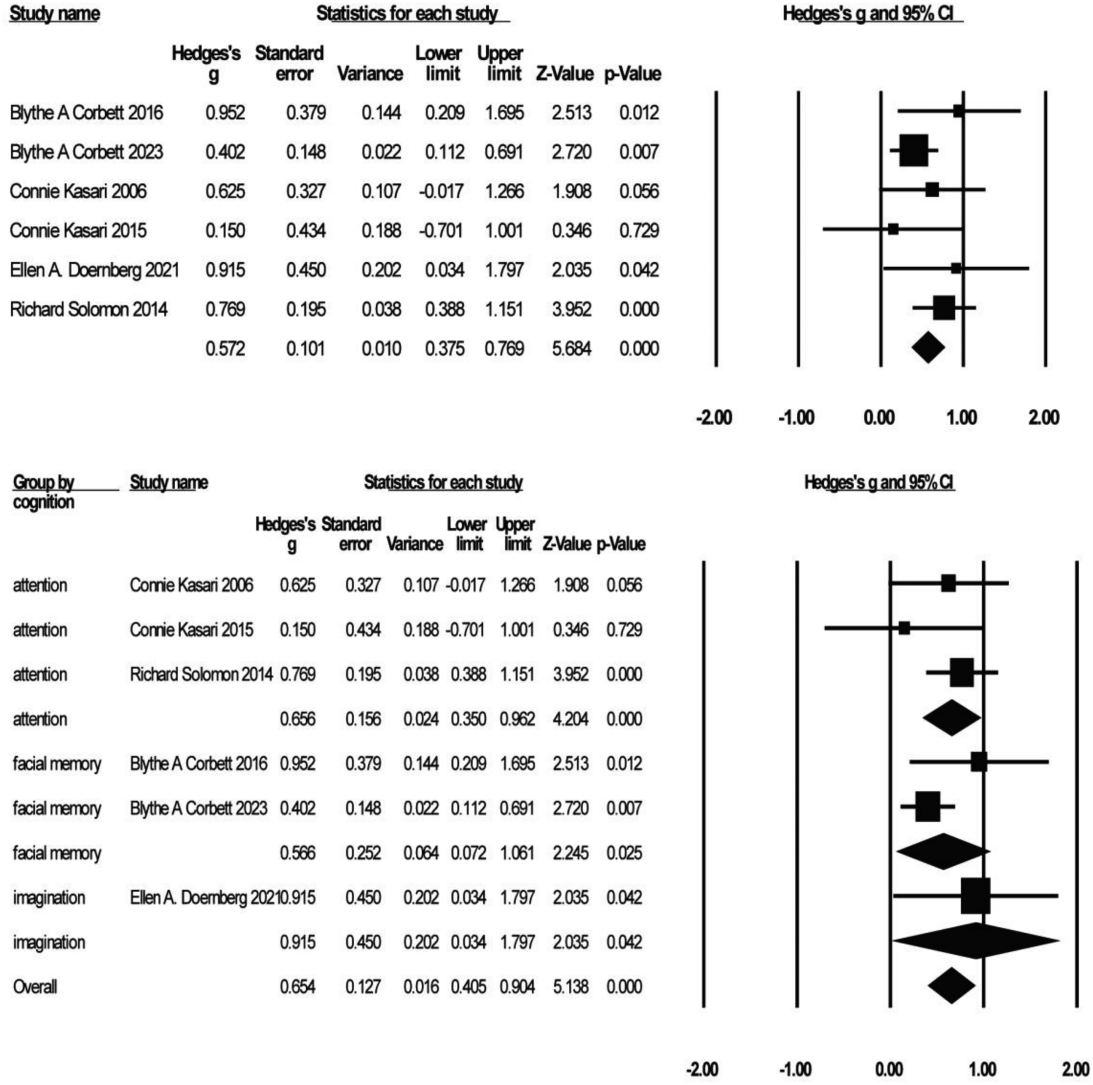
Forest plot of meta-analysis of cognition.

Six studies with 418 participants reported on the effects of GBI on cognitive performance (see Figure 6). Effect sizes ranged from *g* = 0.15 to *g* = 0.95, with positive effects representing cognitive improvement. Compared to the control group, participants who received GBI achieved significantly greater improvement in cognition [*g* = 0.57, *K* = 6, 95% CI (0.38, 0.77), *Z* = 5.68, *p* = 0.00], heterogeneity test (*I*^2^ = 0%), indicating no heterogeneity. Three of the six studies reported levels of attention (27, 70, 74), two reported levels of facial memory (61, 63), and one reported levels of imagination (73), which were analyzed in separate subgroups. The results showed significant improvements in the patients' attention [*g* = 0.66, *K* = 3, 95% CI (0.35, 0.96), *Z* = 4.20, *p* = 0.00] and facial memory [*g* = 0.57, *K* = 2, 95% CI (0.07, 1.06), *Z* = 2.25, *p* = 0.03] levels. There was no heterogeneity for attention (*I*^2^ = 0%) and mild heterogeneity for facial memory (*I*^2^ = 45.47%), but sensitivity analysis could not be performed due to the sample size of only two articles.

#### 3.4.5 Language

**Figure 7:**
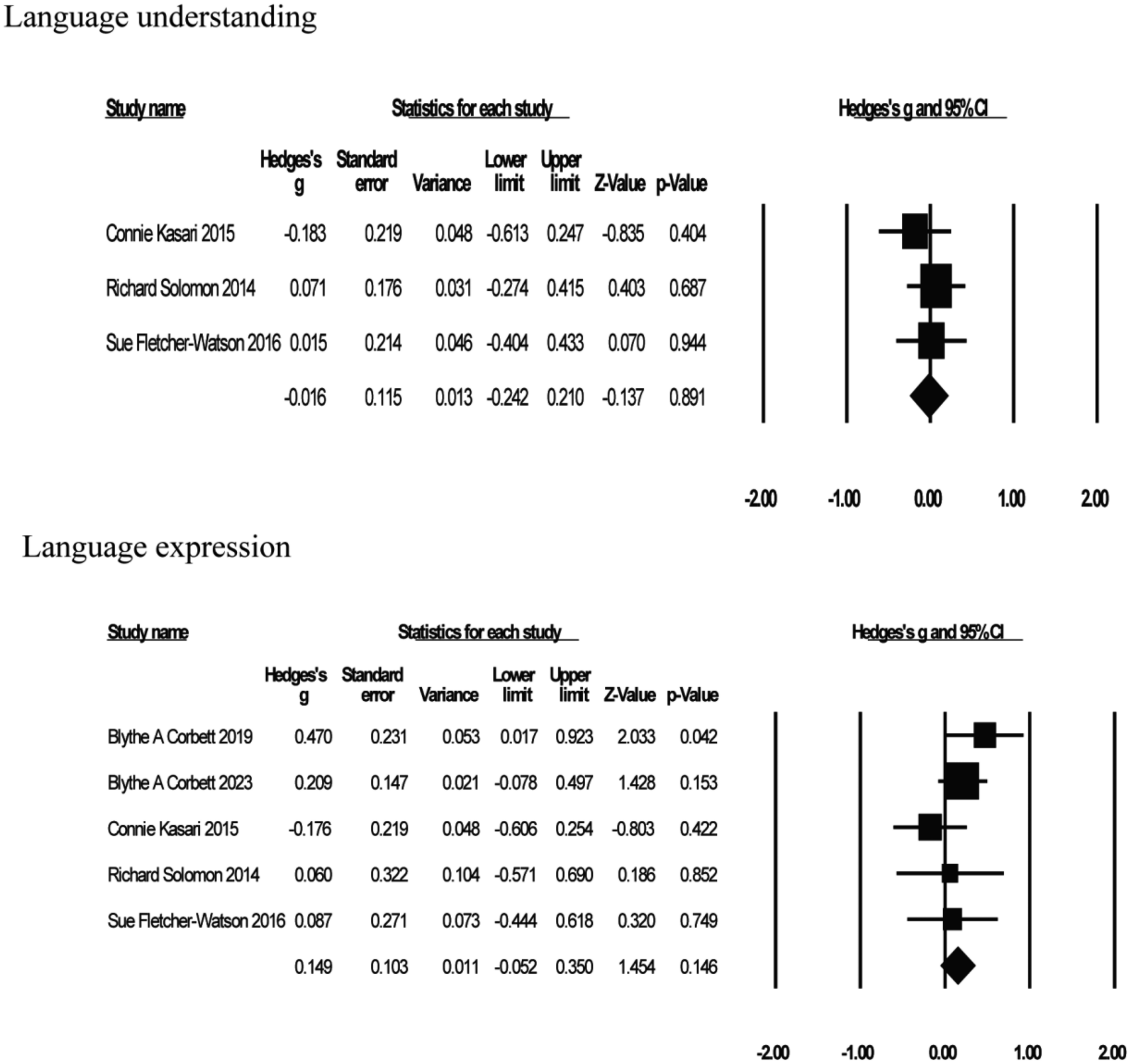
Forest plot of meta-analysis of language understanding and language expression.

Five studies reported on the effects of GBI on the language skills of a child or adolescent with ASD (see Figure 7). Among them, 3 were on language understanding (65, 70, 74) and 5 on language expression (63, 65, 68, 70, 74), and an independent meta-analysis was conducted on language understanding and language expression. Language understanding effect sizes ranged from *g* = −0.18–*g* = 0.07, and language expression effect sizes ranged from *g* = −0.18–*g* = 0.47. The results showed that GBI did not improve language understanding [*g* = −0.02, *K* = 3, 95% CI (−0.24, 0.21), *Z* = −0.14, *p* = 0.89], but it did improve language expression [*g* = 0.15, *K* = 5, 95% CI (−0.05, 0.35), *Z* = 1.45, *p* = 0.15], although neither was significant. The test for heterogeneity in language understanding (*I*^2^ = 0%) indicated no heterogeneity, and language expression (*I*^2^ = 9.58%) showed very little heterogeneity.

#### 3.4.6 Anxiety

**Figure 8:**
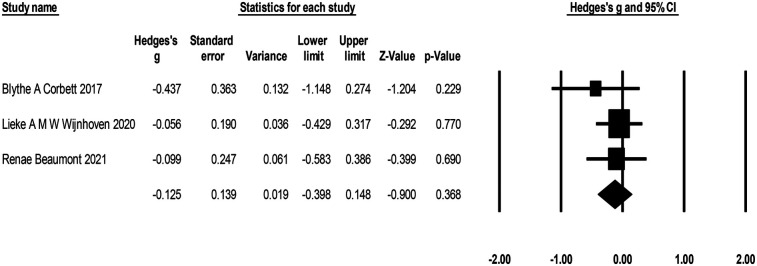
Forest plot of the meta-analysis of anxiety.

Three studies with 203 participants reported levels of anxiety in children and adolescents with autism following GBI treatment (see Figure 8). Effect sizes ranged from *g* = −0.06 to *g* = −0.44, with negative effects representing improvements in anxiety. Compared to the control group, participants who received GBI experienced a reduction in anxiety [*g* = −0.13, *K* = 3, 95% CI (−0.40, 0.15), *Z* = −0.90, *p* = 0.37], but it was not significant. The heterogeneity test (*I*^2^ = 0%) indicated no heterogeneity. No statistical outliers were detected in the sensitivity analysis.

#### 3.4.7 Parenting stress

**Figure 9:**
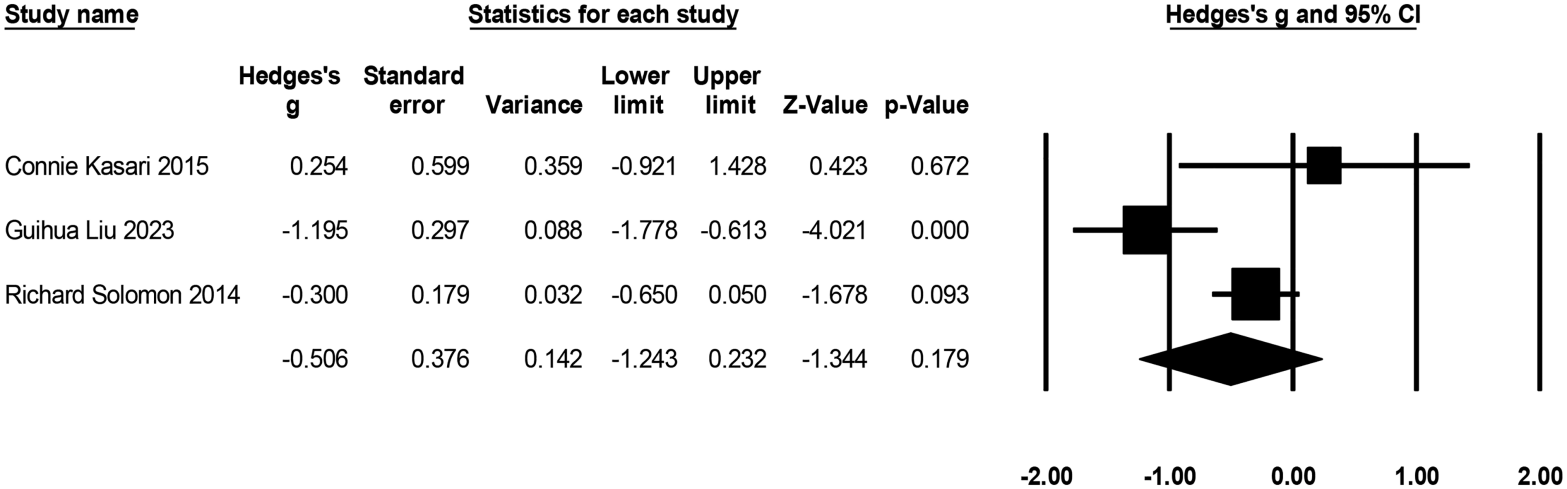
Forest plot of the meta-analysis of parenting stress.

Three studies with 186 participants reported on the effects of play therapy on parenting stress (see Figure 9), with negative effects indicating a reduction in parenting stress. The effect size ranged from *g* = −1.20 to *g* = 0.25. Compared to the control group, play therapy reduced parenting stress [*g* = −0.51, *K* = 3, 95% CI (−1.24, 0.32), *Z* = −1.34, *p* = 0.18], although not significantly. There was high heterogeneity across studies (*I*^2^ = 75.90%). One study (Guihua Liu 2023) was identified as the outlier that contributed most to the between-study heterogeneity, and excluding that piece of research reduced the heterogeneity to 0. The effect sizes were again recombined [*g* = −0.26, 95% CI (−0.59, 0.08), *Z* = −1.49, *p* = 0.14].

### 3.5 Publication bias

**Figure 10:**
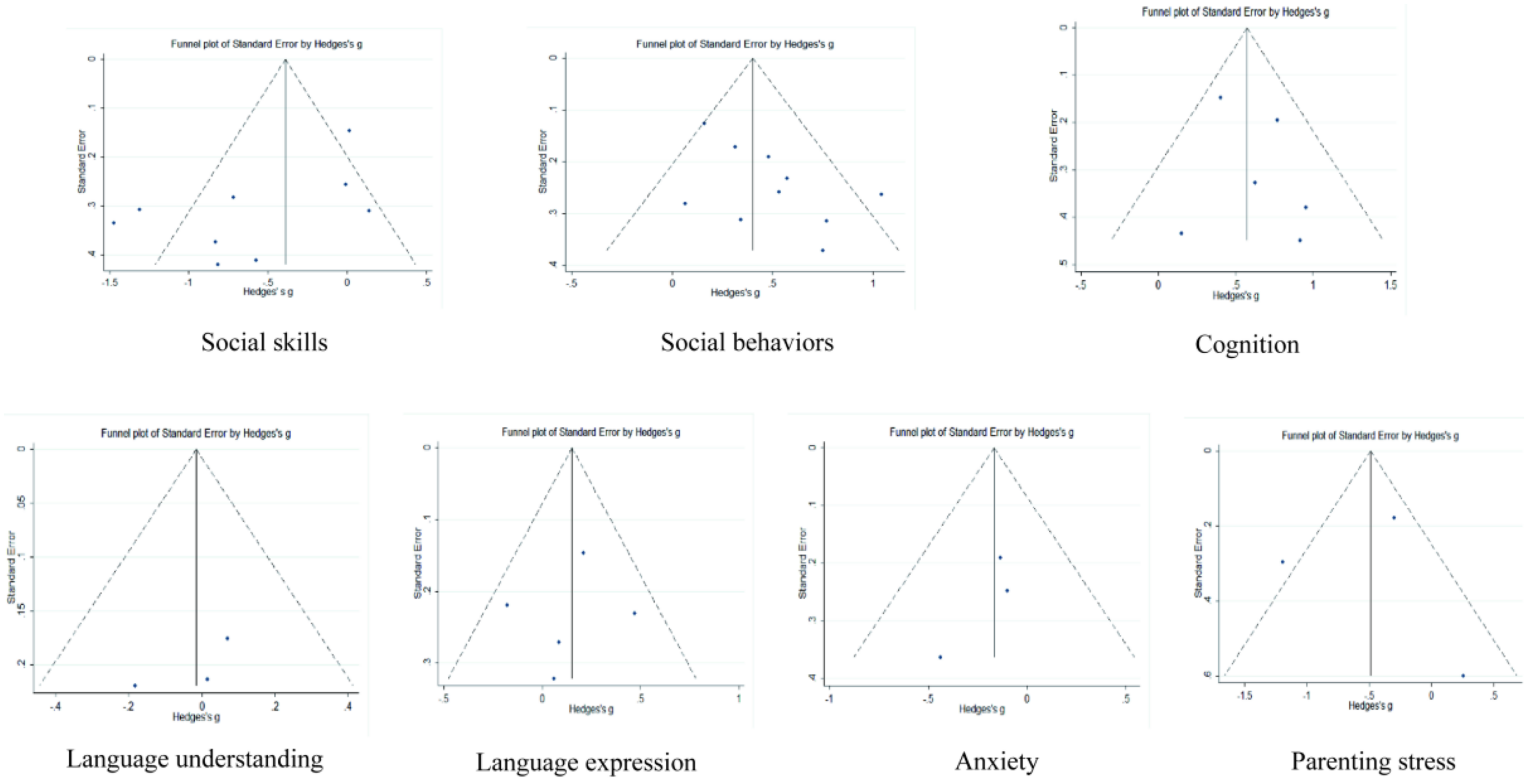
Funnel plot of publication bias.

Publication bias was detected by plotting funnel plots (see Figure 10) as well as Egger's regression test, which visually showed that the funnel plots were roughly symmetrical, and Egger's regression test showed the following: social skills (*p* = 0.056), social behaviors (*p* = 0.055), cognition (*p* = 0.482), language understanding/expression (*p* = 0.429/0.742), anxiety (*p* = 0.369), and parenting stress (*p* = 0.976) were all >0.05. After trim and fill, social behaviors went through 5 iterations, and the 4 effect sizes were imputed on the left side of the funnel plot (i.e., smaller/negative effect sizes), which suggests that the average effect size may be an overestimate of the effect, with a post trim and fill effect size of 0.286. Cognition went through 3 iterations, and the 1 effect size was imputed on the left side of the funnel plot (i.e., smaller/negative effect sizes), indicating that the average effect size may be an overestimate of the effect, with a post trim and fill effect size of 0.543. Regarding parenting Stress, after 2 iterations, 1 effect size was estimated on the left side of the funnel plot (i.e., smaller/negative effect sizes), suggesting that the average effect size may be an underestimation of the effect, and the effect size after clipping was −0.558.

## 4 Discussion

This study is the first comprehensive meta-analysis of GBI for children and adolescents with ASD, including 24 RCTs that quantified the effectiveness of GBI interventions for children and adolescents with ASD. The main findings support significant positive effects of GBI on patients' social skills, social behaviors, and cognition. However, there were no significant effects of GBI treatment on children and adolescents' language skills, anxiety, or parental parenting stress.

### 4.1 Effects of GBI on socialization in children and adolescents with ASD

We evaluated the social skills of individuals with ASD by SRS scores, and the total effect of GBI on the improvement of participants' SRS scores was significant (*g* = −0.59, *p* = 0.004), which shows that GBI significantly improved the social skills of children and adolescents with ASD. GBI is a patient-centered, nondirective, unstructured, and free-form therapy (77) that creates opportunities for children to express their feelings, explore relationships, and describe their experiences (78), which in turn promotes skills such as concentration/attention, efficiency, organizational skills, dexterity, and working memory (79). In the subgroup analyses, the improvement effect was significant in children (*g* = −0.81, *p* = 0.001) but smaller and not statistically significant in adolescents (*g* = −0.16, *p* = 0.52). This result may be attributed to the fact that childhood is a period of rapid brain development and basic acquisition of cognition (i.e., working memory, attention, and inhibitory control) compared to adolescence, their brains and behaviors are more malleable (80, 81). ADOS-2, as a standardized ASD diagnostic tool with high specificity and sensitivity, is the gold standard for ASD diagnosis (82). The ADOS-2 outcome showed improvement in scores on the social affect domain (*g* = −0.11) but not on the restricted and repetitive behavior domain (*g* = 0.05), but the strength of the evidence for the results was low due to the small sample size as well as the heterogeneity of the studies. Two studies reported completely opposite ADOS-2 outcomes, possibly due to differences in the duration of intervention as well as treatment measures. The Gerald Mahoney study was conducted over a 12-month play session (66), whereas the Sue Fletcher-Watson study was conducted over a 2-month video game therapy (65), and the skills learned in the 2D animated game were challenging to generalize to real-world scenarios (83). GBI significantly improved social behaviors in children with ASD (*g* = 0.45, *p* = 0.00), and the results of a past clinical study similarly validated this finding. This study found that as GBI progressed, children slowly made more eye contact, approached the play therapist more frequently, and showed a desire to participate and build connections (84). GBI meets the child's developmental level, in which the child expresses him/herself in the most comfortable way and can enjoy the feeling of complete acceptance, thus establishing safe social relationships with a high potential for generalization to social interactions in nontherapeutic environments (26). However, there was mild to moderate heterogeneity in our study, and sensitivity analyses excluding one study (67) reduced the heterogeneity to close to zero and reduced the effect size, suggesting that this study may have amplified the effect size. The result may be because parents were both the intervention-delivery agents and evaluators, making them more susceptible to responder bias.

### 4.2 Effects of GBI on cognition and language in children and adolescents with ASD

At the cognitive level, GBI significantly improved attention and facial memory in children and adolescents with ASD. Attention and facial memory are critical for building and maintaining social relationships, and they tend to represent more social behaviors as well as fewer ASD symptoms (85–88). Patients are more concentrated when they are free to choose games of interest during play. Additionally, parents report that their children are able to play and concentrate for longer periods when playing app-based games (65) and that their eyes are more sensitive to cues in serious games (25). The use of role-playing treatments in both studies targeting facial memory improvement showed that during active role-playing, children can experience a variety of elements that contribute to increased awareness and interest in social stimuli, increased facial salience, and improved face memory (61). After the GBI intervention, although not significantly, the child's language expression was improved. In the play process, children's attention to and engagement with others increases, improving mutual verbal communication (63). However, our study found suboptimal improvements in language understanding, demonstrating that while most children's language skills are first receptive and then expressive in manner, children with severe ASD may learn words by saying them before understanding them (89).

### 4.3 Effects of GBI on anxiety in children and adolescents with ASD and on parental parenting stress

Three studies reported improvements in patient anxiety, yet the overall effect was not significant. Improvements in anxiety symptoms can be explained by the overall positive impact of play on children's mental health (90, 91). Children can project their anxieties into play elements and cope with these anxieties through relaxation during play (Ferguson & Olson, 2013). However, due to the small sample size involved in the analysis, statistical significance was not reached at this time. In addition, we conducted an analysis of parental stress, and there is no doubt that raising a child with ASD increases parental parenting stress (92), GBI designed for children with ASD typically focuses not only on the child's social, emotional, and behavioral development but also aims to improve family relationships and parent-child interactions. While play therapy can have positive effects on the child, it also helps reduce the behavioral challenges parents encounter in daily caregiving. When parents are able to manage their child's behavior more effectively, reduce frustration, and enhance their confidence, their stress levels tend to decrease (66). Although improvements in the child's symptoms following GBI treatment may alleviate parenting stress to some extent, our study did not find a significant reduction in parenting stress. This result may be attributed to the limitations of the sample size or variations in the level of parental involvement in the treatment.

### 4.4 Advantages of GBI and clinical application recommendations

GBI have shown significant potential in the treatment of individuals with ASD due to their interactivity and flexibility (26, 93). Compared to traditional interventions, GBI not only improve the core symptoms of ASD but also effectively utilize the individual's unique strengths, creating personalized and engaging intervention experiences that promote comprehensive development in cognitive, emotional, and social abilities (26). GBI leverage the interests of individuals with ASD to stimulate their motivation to engage (39). Individuals with autism often demonstrate intense focus in specific areas of interest. By designing game activities that align with these interests, participation can be significantly enhanced. For example, if an individual is interested in transportation vehicles, puzzles or role-playing games designed around this interest can not only increase engagement but also provide opportunities for social interaction. The task-oriented nature of the games can also improve cognitive abilities, particularly in areas such as systematic thinking and problem-solving. Many individuals with autism have a natural preference for rules and structure. Through structured games, they can develop their logical thinking and strategic planning skills while adhering to rules. Moreover, GBI can also help improve sensory regulation abilities in individuals with ASD. ASD individuals often experience difficulties in sensory processing, but they may have heightened sensitivity to certain sensory inputs, such as visual or auditory stimuli (3). Through digital platforms (such as VR or augmented reality technology) and multisensory interactive devices, games can provide personalized sensory support, helping individuals gradually adapt to complex sensory stimuli (34, 35). This technological intervention not only effectively enhances the intervention outcomes but also provides a safe learning environment, reducing overreactions to external stimuli and improving sensory integration abilities (35). In terms of social interaction and emotional expression, GBI have also shown significant advantages. Individuals with ASD often face difficulties in social interactions, and through cooperative games or role-playing games, they can learn and practice social skills in a non-threatening environment. Role-playing games, in particular, provide an opportunity to simulate social situations, allowing children to understand and adapt to social rules while playing different roles (63, 68, 75). At the same time, these games enhance individuals' self-confidence and emotional expression abilities through immediate feedback and the accumulation of a sense of achievement, thereby contributing to their emotional regulation and social interactions in real life (75). GBI also have a high degree of personalization, allowing them to be tailored to the interests and needs of each individual with autism. Through personalized design, the intervention content can better align with the individual's cognitive level, social needs, and emotional expression, thereby more effectively promoting overall development. Particularly in the treatment of ASD, intervention design should fully consider the individual's strengths, such as potential in task orientation and detail analysis, helping them achieve a sense of accomplishment in areas of interest, which in turn enhances their overall abilities.

GBI in clinical applications should follow the principle of precise assessment. Before starting the intervention, standardized assessment tools should be used to systematically understand the strengths and needs of each individual with ASD, providing a scientific basis for the design of game activities. This assessment process not only helps the treatment team develop personalized intervention plans but also provides a basis for adjusting the content of the games. During the intervention, game-based approaches need to collaborate with a multidisciplinary team to ensure the effectiveness of the intervention. Psychologists, therapists, educators, and parents should all be involved, providing professional support and feedback. Therapists can adjust the game content based on observations of the individual's performance in the games, ensuring that it both stimulates the individual's interest and effectively promotes their development. The use of technology is also an important complement to GBI. Digital games, virtual reality(VR), and augmented reality(AR) technologies offer more innovative possibilities for interventions (65, 67, 79, 94). These technologies not only provide personalized game content based on individual needs but also allow real-time data recording and feedback mechanisms to monitor the effects of interventions and dynamically adjust them. Therefore, therapists can precisely assess the progress of interventions through technological means, adjusting intervention strategies in a timely manner to improve the accuracy and effectiveness of treatment. Active involvement of the family is equally crucial in GBI (66, 95). When parents understand the design intentions and implementation process of the games, they can extend the intervention into daily life, improving the consistency and effectiveness of the intervention. Providing training and support to parents, enabling them to guide and support the child's play activities in the home environment, is key to enhancing the intervention's effectiveness (96). Additionally, regular communication between therapists and parents can ensure the proper implementation of intervention measures, further promoting progress in children with ASD. GBI provide a therapeutic platform for individuals with ASD that both leverages their strengths and improves core symptoms. Through precise assessment, personalized design, multidisciplinary collaboration, and the use of technological tools, GBI can not only enhance individuals' engagement and effectiveness but also promote the comprehensive development of their social, emotional, and cognitive abilities.

The practical implementation of GBI encompasses clinical rehabilitation centers, schools, and home settings. In clinical rehabilitation centers, professional play therapists can utilize structured play training to enhance the social interaction skills of children with ASD. For instance, social story games can guide them in understanding social norms, while cooperative games (e.g., LEGO Therapy) can strengthen teamwork abilities. Additionally, the application of VR and AR technologies can create controlled and safe environments, allowing ASD children to engage in immersive social interactions and improve their real-world adaptability. Although these technologies require a higher initial investment than traditional intervention methods, they offer personalized training programs, reduce labor costs, and enhance intervention efficiency in the long run. In school environments, educators can employ gamification strategies to increase the learning motivation and social adaptability of ASD children. For example, embedding a point-based reward system in the classroom can encourage them to initiate greetings or participate in group activities. Role-playing games can further help children practice real-life social scenarios, fostering empathy and problem-solving skills. School-based intervention programs generally offer high accessibility and can be implemented within existing educational resources, ensuring cost-effectiveness and broader benefits for ASD children. In home settings, parents can facilitate communication through parent-child interactive games (e.g., board games) and create more opportunities for natural interactions in daily life, thereby helping ASD children develop social skills in a familiar environment. Traditional tabletop games or structured training approaches are cost-effective and easy to implement. Moreover, integrating mobile applications (such as Mindreading or Social Express) can further enhance intervention effectiveness. Although the efficacy of GBI in ASD interventions has been supported by emerging evidence, further optimization of individualized game-based programs is necessary to align with the specific interests and developmental levels of ASD children. Additionally, enhancing the training of parents and educators to effectively guide ASD children in engaging with games is a crucial strategy for ensuring the sustainability of interventions. Overall, by improving accessibility and cost-effectiveness, GBI can play a significant role across various settings, providing comprehensive and personalized support for ASD children while offering practical guidance for professionals, educators, and caregivers.

## 5 Limitations

The results of this meta-analysis need to be interpreted in the context of several limitations. Firstly, although the ADOS is considered the gold standard for evaluating ASD, the studies included in our analysis did not report total ADOS scores, and the limited number of studies made it impossible to conduct a comprehensive assessment of the overall impact on ASD levels. Secondly, while this study included both children and adolescents with ASD, there were noticeably fewer studies on adolescents compared to children. However, the research suggests that during adolescence, when social demands exceed social skills, the symptoms of autism may become more pronounced (13). Future research should further focus on the effects of GBI on adolescents with ASD to prevent continued impairments into adulthood. Thirdly, through the trim-and-fill method, we found evidence of potential publication bias in the areas of social behavior, cognition, and parenting stress, which suggests that the true effect might be lower than what we have reported. Nevertheless, overall, the results of the current meta-analysis indicate that GBI is valuable for children and adolescents with ASD. Fourthly, play therapy must be delivered by trained play therapists following established therapeutic protocols. However, current research in this area often lacks consistency in how “play therapy” is implemented, with many studies involving interventionists who do not meet the qualifications of certified play therapists. To enhance the effectiveness and reliability of play therapy interventions, future research should adopt more standardized protocols. It is essential that practitioners delivering play therapy undergo formal training to ensure fidelity to the intervention model. This will not only improve the quality of the research but also contribute to the broader development and refinement of the field of play therapy. Fifthly, the diagnosis and treatment of ASD are influenced by age and cultural factors (1, 97). Children of different ages exhibit variations in social, language, and behavioral expressions. Early interventions in infancy focus on language and social skills, while school-age children focus on social skills training and emotional regulation. In adolescence, the emphasis shifts to emotional understanding and self-regulation. Moreover, cultural background also affects the recognition of autism symptoms and intervention methods, as different cultures may have varying expectations of behavior, which in turn can influence treatment outcomes. Therefore, treatments should take cultural differences into account and develop individualized plans (97, 98), For example, in GBI, structured play can promote interaction in early childhood, role-playing can enhance social abilities in school-age children, and complex games can improve emotional management and social skills in adolescence. Finally, RCTs are widely regarded as the gold standard in clinical research, providing valuable insights into the effectiveness of interventions. All studies included in this article are RCTs. However, when applied to the ASD population, RCTs have limitations in fully capturing individual differences. ASD is a spectrum disorder, and individuals exhibit a wide range of abilities, challenges, and responses to interventions. Averaging data from a diverse population may obscure cases where certain interventions are effective for some individuals but not for others. This oversimplification of treatment outcomes may overlook subtle variations in intervention effects. In contrast, single-case design (SCD) studies offer a more personalized approach to intervention evaluation by conducting in-depth analysis of individual participants. By tracking changes in individual responses over time, SCD can capture individual differences and subtle effects of interventions, making it particularly useful for understanding personalized treatments such as play therapy. Therefore, for future research, we recommend adopting a mixed-methods approach that combines RCTs and single-case designs, ensuring conclusions that are broadly applicable while also providing an in-depth analysis of the effects of individualized interventions, thus offering a comprehensive understanding of the diversity and personalized needs in ASD interventions.

## 6 Conclusion

Our analysis indicates that GBI can effectively improve social skills, social behavior, and cognitive abilities in children and adolescents with ASD. However, its effects on language skills, anxiety, and parenting stress were not statistically significant. While these findings highlight the potential benefits of GBI, the current meta-analysis is limited by sample size and study quality, which may affect the strength of the evidence.

To optimize the clinical application of GBI, future research should focus on developing structured implementation guidelines, including standardized assessment tools to tailor interventions based on individual strengths and needs. Furthermore, multidisciplinary collaboration among therapists, educators, and caregivers is essential to ensure intervention fidelity and effectiveness. Given that individuals with ASD often exhibit strong motivation when engaging in activities aligned with their interests, future GBI should incorporate personalized content and task-oriented elements to enhance engagement, cognitive development, and social skills. In practical settings, GBI should be integrated into clinical rehabilitation centers, schools, and home-based programs to maximize accessibility and real-world impact. In clinical environments, interventions should be delivered by certified play therapists to ensure treatment efficacy. Schools can adopt gamification strategies to increase engagement in social skills training, while families can incorporate interactive games into daily routines to extend therapeutic benefits. Additionally, training programs for caregivers and educators should be implemented to ensure proper guidance and long-term sustainability of interventions. By refining intervention protocols, expanding research methodologies, and fostering interdisciplinary collaboration, GBI can become a scalable, personalized, and effective intervention, ultimately enhancing the overall well-being of individuals with ASD.

## Data Availability

The original contributions presented in the study are included in the article/Supplementary Material, further inquiries can be directed to the corresponding authors.

## References

[1] Hirota T King BH. Autism spectrum disorder: a review. JAMA. (2023) 329:157–68. 10.1001/jama.2022.23661 36625807

[2] Khan NZ Gallo LA Arghir A Budisteanu B Budisteanu M Dobrescu I Autism and the grand challenges in global mental health. Autism Res. (2012) 5:156–9. 10.1002/aur.1239 22605618

[3] Mottron L Dawson M Soulières I Hubert B Burack J. Enhanced perceptual functioning in autism: an update, and eight principles of autistic perception. J Autism Dev Disord. (2006) 36:27–43. 10.1007/s10803-005-0040-7 16453071

[4] Braconnier ML Siper PM. Neuropsychological assessment in autism spectrum disorder. Curr Psychiatry Rep. (2021) 23:63. 10.1007/s11920-021-01277-1 34331144 PMC8324442

[5] Courchesne V Langlois V Gregoire P St-Denis A Bouvet L Ostrolenk A Interests and strengths in autism, useful but misunderstood: a pragmatic case-study. Front Psychol. (2020) 11:569339. 10.3389/fpsyg.2020.569339 33123051 PMC7573358

[6] Elsabbagh M Divan G Koh YJ Kim YS Kauchali S Marcín C Global prevalence of autism and other pervasive developmental disorders. Autism Res. (2012) 5:160–79. 10.1002/aur.239 22495912 PMC3763210

[7] Howlin P Magiati I. Autism spectrum disorder: outcomes in adulthood. Curr Opin Psychiatry. (2017) 30:69–76. 10.1097/yco.0000000000000308 28067726

[8] Ameis SH Kassee C Corbett-Dick P Cole L Dadhwal S Lai MC Systematic review and guide to management of core and psychiatric symptoms in youth with autism. Acta Psychiatr Scand. (2018) 138:379–400. 310.1111/acps.12918 29904907 10.1111/acps.12918

[9] Mutluer T Aslan Genç H Özcan Morey A Yapici Eser H Ertinmaz B Can M Population-based psychiatric comorbidity in children and adolescents with autism spectrum disorder: a meta-analysis. Front Psychiatry. (2022) 13:856208. 10.3389/fpsyt.2022.856208 35693977 PMC9186340

[10] Simonoff E Pickles A Charman T Chandler S Loucas T Baird G. Psychiatric disorders in children with autism spectrum disorders: prevalence, comorbidity, and associated factors in a population-derived sample. J Am Acad Child Adolesc Psychiatry. (2008) 47:921–9. 10.1097/CHI.0b013e318179964f 18645422

[11] Ung D Wood JJ Ehrenreich-May J Arnold EB Fuji C Renno P Clinical characteristics of high-functioning youth with autism spectrum disorder and anxiety. Neuropsychiatry. (2013) 3(2):10–2217. 10.2217/npy.13.9 PMC3808966 24179485

[12] Lord C Elsabbagh M Baird G Veenstra-Vanderweele J. Autism spectrum disorder. Lancet. (2018) 392:508–20. 10.1016/s0140-6736(18)31129-2 30078460 PMC7398158

[13] Picci G Scherf KS. A two-hit model of autism: adolescence as the second hit. Clin Psychol Sci. (2015) 3:349–71. 10.1177/2167702614540646 26609500 PMC4655890

[14] Sandbank M Bottema-Beutel K Crowley S Cassidy M Dunham K Feldman JI Project AIM: autism intervention meta-analysis for studies of young children. Psychol Bull. (2020) 146:1–29. 10.1037/bul0000215 31763860 PMC8783568

[15] Schröder CM Florence E Dubrovskaya A Lambs B Stritmatter P Vecchionacci V Une approche d’intervention précoce pour les troubles du spectre autistique. Neuropsychiatr Enfance Adolesc. (2015) 63:279–87. 10.1016/j.neurenf.2015.04.001

[16] Flippin M Reszka S Watson LR. Effectiveness of the Picture Exchange Communication System (PECS) on communication and speech for children with autism spectrum disorders: a meta-analysis. Am J Speech Lang Pathol. (2010) 19(2):178–95. 10.1044/1058-0360(2010/09-0022) 20181849

[17] Voss C Schwartz J Daniels J Kline A Haber N Washington P Effect of wearable digital intervention for improving socialization in children with autism spectrum disorder: a randomized clinical trial. JAMA Pediatr. (2019) 173:446–54. 10.1001/jamapediatrics.2019.0285 30907929 PMC6503634

[18] Brignell A Chenausky KV Song H Zhu J Suo C Morgan AT. Communication interventions for autism spectrum disorder in minimally verbal children. Cochrane Database Syst Rev. (2018) 11(11):CD012324. 10.1002/14651858.CD012324.pub2 30395694 PMC6516977

[19] Law J Dennis J Charlton J. Speech and language therapy interventions for children with primary speech and/or language disorders. Cochrane Database Syst Rev. (2017) 2017(1):CD012490. 10.1002/14651858.CD012490 PMC8407295 12918003

[20] Hébert ML Kehayia E Prelock P Wood-Dauphinee S Snider L. Does occupational therapy play a role for communication in children with autism spectrum disorders? Int J Speech Lang Pathol. (2014) 16:594–602. 10.3109/17549507.2013.876665 24460071

[21] Novak I. Effectiveness of occupational therapy intervention for children with disabilities: systematic review. Dev Med Child Neurol. (2016) 58:29–30. Conference Abstract. doi: 10.1111/dmcn.13069 26411643

[22] Andanson J Pourre F Maffre T Raynaud JP. Social skills training groups for children and adolescents with asperger syndrome: a review. Arch Pediatr. (2011) 18:589–96. 10.1016/j.arcped.2011.02.019 21458972

[23] Freitag C. Social skills training in high-functioning autism spectrum disorder. Eur Child Adolesc Psychiatry. (2013) 22:S177. 10.1007/s00787-013-0423-9

[24] Gates JA Kang E Lerner MD. Efficacy of group social skills interventions for youth with autism spectrum disorder: a systematic review and meta-analysis. Clin Psychol Rev. (2017) 52:164–81. 10.1016/j.cpr.2017.01.006 28130983 PMC5358101

[25] Griffin JW Geier CF Smyth JM Suzanne Scherf K. Improving sensitivity to eye gaze cues in adolescents on the autism spectrum using serious game technology: a randomized controlled trial. JCPP Adv. (2021) 1:e12041. 10.1002/jcv2.12041 36643718 PMC9835110

[26] Schottelkorb AA Swan KL Ogawa Y. Intensive child-centered play therapy for children on the autism spectrum: a pilot study. J Couns Dev. (2020) 98:63–73. 10.1002/jcad.12300

[27] Kasari C Freeman S Paparella T. Joint attention and symbolic play in young children with autism: a randomized controlled intervention study. J Child Psychol Psychiatry. (2006) 47:611–20. 10.1111/j.1469-7610.2005.01567.x 16712638

[28] Kasari C Paparella T Freeman S Jahromi LB. Language outcome in autism: randomized comparison of joint attention and play interventions. J Consult Clin Psychol. (2008) 76:125–37. 10.1037/0022-006x.76.1.125 18229990

[29] Morrier MJ Ziegler SMT. I wanna play too: factors related to changes in social behavior for children with and without autism spectrum disorder after implementation of a structured outdoor play curriculum. J Autism Dev Disord. (2018) 48:2530–41. 10.1007/s10803-018-3523-z 29488050

[30] Chang YC Shih W Landa R Kaiser A Kasari C. Symbolic play in school-aged minimally verbal children with autism spectrum disorder. J Autism Dev Disord. (2018) 48:1436–45. 10.1007/s10803-017-3388-6 29170936

[31] Dell'Angela L Zaharia A Lobel A Vico Begara O Sander D Samson AC. Board Games on Emotional Competences for School-Age Children. Games Health J. (2020) 9(3):187–96. 10.1089/g4h.2019.0050 32053027

[32] Giannopulu I Pradel G. Multimodal interactions in free game play of children with autism and a mobile toy robot. NeuroRehabilitation. (2010) 27:305–11. 10.3233/nre-2010-0613 21160119

[33] Sautter RA LeBlanc LA Gillett JN. Using free operant preference assessments to select toys for free play between children with autism and siblings. Res Autism Spectr Disord. (2008) 2:17–27. 10.1016/j.rasd.2007.02.001

[34] Zhang M Ding H Naumceska M Zhang Y. Virtual reality technology as an educational and intervention tool for children with autism spectrum disorder: current perspectives and future directions. Behav Sci (Basel). (2022) 12(5):138. 10.3390/bs12050138 35621435 PMC9137951

[35] Kuhlthau KA Luberto CM Traeger L Millstein RA Perez GK Lindly OJ A virtual resiliency intervention for parents of children with autism: a randomized pilot trial. J Autism Dev Disord. (2020) 50:2513–26. 10.1007/s10803-019-03976-4 30900195 PMC6864241

[36] LeGoff DB. Use of LEGO© as a therapeutic medium for improving social competence. J Autism Dev Disord. (2004) 34:557–71. 10.1007/s10803-004-2550-0 15628609

[37] Guénoun T Tiberghien C Juteau A. Videodrama: cartoon-based therapeutic mediation for children with autism spectrum disorders. Neuropsychiatr Enfance Adolesc. (2021) 69:221–7. 10.1016/j.neurenf.2021.05.004

[38] Landreth GL. Play Therapy: The Art of the Relationship. New York: Routledge (2012).

[39] Fiss AL Håkstad RB Looper J Pereira SA Sargent B Silveira J Embedding play to enrich physical therapy. Behav Sci. (2023) 13(6):440. 10.3390/bs13060440 37366692 PMC10295001

[40] Association and Therapy fP. Available online at: https://www.a4pt.org (accessed October 21, 2019).

[41] Cohen E Gadassi R. The function of play for coping and therapy with children exposed to disasters and political violence. Curr Psychiatry Rep. (2018) 20:31. 10.1007/s11920-018-0895-x 29623498

[42] Chang YC Locke J. A systematic review of peer-mediated interventions for children with autism spectrum disorder. Res Autism Spectr Disord. (2016) 27:1–10. 10.1016/j.rasd.2016.03.010 27807466 PMC5087797

[43] Wolfberg P DeWitt M Young GS Nguyen T. Integrated play groups: promoting symbolic play and social engagement with typical peers in children with ASD across settings. J Autism Dev Disord. (2015) 45(3):830–45. 10.1007/s10803-014-2245-0 25231289

[44] Bernardini S Porayska-Pomsta K Smith TJ. ECHOES: an intelligent serious game for fostering social communication in children with autism. Inf Sci (Ny). (2014) 264:41–60. 10.1016/j.ins.2013.10.027

[45] Davidson D Stagnitti K. The process of learn to play therapy with parent-child dyads with children who have autism spectrum disorder. Aust Occup Ther J. (2021) 68:419–33. 10.1111/1440-1630.12751 34312879

[46] Hu X Zheng Q Lee GT. Using peer-mediated LEGO® play intervention to improve social interactions for Chinese children with autism in an inclusive setting. J Autism Dev Disord. (2018) 48:2444–57. 10.1007/s10803-018-3502-4 29453705

[47] Tuerk S Korfmacher AK Gerger H Van der Oord S Christiansen H. Interventions for ADHD in childhood and adolescence: a systematic umbrella review and meta-meta-analysis. Clin Psychol Rev. (2023) 102:102271. 10.1016/j.cpr.2023.102271 37030086

[48] Werner-Seidler A Perry Y Calear AL Newby JM Christensen H. School-based depression and anxiety prevention programs for young people: a systematic review and meta-analysis. Clin Psychol Rev. (2017) 51:30–47. 10.1016/j.cpr.2016.10.005 27821267

[49] Higgins JP Altman DG Gøtzsche PC Jüni P Moher D Oxman AD The cochrane collaboration’s tool for assessing risk of bias in randomised trials. Br Med J. (2011) 343:d5928. 10.1136/bmj.d5928 22008217 PMC3196245

[50] Moher D Liberati A Tetzlaff J Altman DG, Prisma Group. Preferred reporting items for systematic reviews and meta-analyses: the PRISMA statement. PLoS Med. (2009) 6:e1000097. 10.1371/journal.pmed.1000097 19621072 PMC2707599

[51] Hedges LV Olkin I. Statistical Methods for Meta-analysis. San Diego, California: Academic Press Inc. (1985).

[52] Cohen J. A power primer. Psychol Bull. (1992) 112:155–9. 10.1037//0033-2909.112.1.155 19565683

[53] Huedo-Medina TB Sánchez-Meca J Marín-Martínez F Botella J. Assessing heterogeneity in meta-analysis: Q statistic or I2 index? Psychol Methods. (2006) 11:193–206. 10.1037/1082-989x.11.2.193 16784338

[54] Fernández-Castilla B Jamshidi L Declercq L Beretvas SN Onghena P Van den Noortgate W. The application of meta-analytic (multi-level) models with multiple random effects: a systematic review. Behav Res Methods. (2020) 52:2031–52. 10.3758/s13428-020-01373-9 32162276

[55] Egger M Davey Smith G Schneider M Minder C. Bias in meta-analysis detected by a simple, graphical test. Br Med J. (1997) 315:629–34. 10.1136/bmj.315.7109.629 9310563 PMC2127453

[56] Egger M Smith GD. Bias in location and selection of studies. Br Med J. (1998) 316:61–6. 10.1136/bmj.316.7124.61 9451274 PMC2665334

[57] Duval S Tweedie R. Trim and fill: a simple funnel-plot-based method of testing and adjusting for publication bias in meta-analysis. Biometrics. (2000) 56:455–63. 10.1111/j.0006-341x.2000.00455.x 10877304

[58] Li GK Ge P Liu GH Huang XX Lu GB Wang YX Clinical effect of integrated sandplay therapy in children with asperger syndrome. Zhongguo Dang Dai Er Ke Za Zhi. (2019) 21:234–8. 10.7499/j.issn.1008-8830.2019.03.009 30907346 PMC7389368

[59] Liu G Huang L Qian Q Wang YX Ge P. Curative effect of progressively integrated sandplay therapy on core symptoms and sleep management in preschool children with mild-to-moderate autism spectrum disorder. Chin J Contemp Pediatr. (2019) 21:743–8. 10.7499/j.issn.1008-8830.2019.08.002 PMC7389896 31416496

[60] Liu G Chen Y Ou P Huang L Qian Q Wang Y Effects of parent-child sandplay therapy for preschool children with autism spectrum disorder and their mothers: a randomized controlled trial. J Pediatr Nurs. (2023) 71:6–13. 10.1016/j.pedn.2023.02.006 36947897

[61] Corbett BA Key AP Qualls L Fecteau S Newsom C Coke C Improvement in social competence using a randomized trial of a theatre intervention for children with autism spectrum disorder. J Autism Dev Disord. (2016) 46:658–72. 10.1007/s10803-015-2600-9 26419766 PMC5633031

[62] Penev Y Dunlap K Husic A Hou C Washington P Leblanc E A mobile game platform for improving social communication in children with autism: a feasibility study. Appl Clin Inform. (2021) 12:1030–40. 10.1055/s-0041-1736626 34788890 PMC8598393

[63] Corbett BA White S Lerner M Preacher KJ Klemencic ME Simmons GL Peers, play, and performance to build social salience in autistic youth: a multisite randomized clinical trial. J Consult Clin Psychol. (2023) 91:411–25. 10.1037/ccp0000821 37199977 PMC10330829

[64] So WC Cheng CH Lam WY Huang Y Ng KC Tung HC A robot-based play-drama intervention may improve the joint attention and functional play behaviors of Chinese-speaking preschoolers with autism spectrum disorder: a pilot study. J Autism Dev Disord. (2020) 50:467–81. 10.1007/s10803-019-04270-z 31655965

[65] Fletcher-Watson S Petrou A Scott-Barrett J Dicks P Graham C O’Hare A A trial of an iPad™ intervention targeting social communication skills in children with autism. Autism. (2016) 20:771–82. 10.1177/1362361315605624 26503990 PMC5015758

[66] Mahoney G Solomon R. Mechanism of developmental change in the PLAY project home consultation program: evidence from a randomized control trial. J Autism Dev Disord. (2016) 46:1860–71. 10.1007/s10803-016-2720-x 26830414

[67] Beaumont R Walker H Weiss J Sofronoff K. Randomized controlled trial of a video gaming-based social skills program for children on the autism spectrum. J Autism Dev Disord. (2021) 51:3637–50. 10.1007/s10803-020-04801-z 33389304 PMC7778851

[68] Corbett BA Ioannou S Key AP Coke C Muscatello R Vandekar S Treatment effects in social cognition and behavior following a theater-based intervention for youth with autism. Dev Neuropsychol. (2019) 44:481–94. 10.1080/87565641.2019.1676244 31589087 PMC6818093

[69] Kent C Cordier R Joosten A Wilkes-Gillan S Bundy A. Can I learn to play? Randomized control trial to assess effectiveness of a peer-mediated intervention to improve play in children with autism spectrum disorder. J Autism Dev Disord. (2021) 51:1823–38. 10.1007/s10803-020-04671-5 32870416

[70] Solomon R Van Egeren LA Mahoney G Huber Q Melissa S Zimmerman P. PLAY Project home consultation intervention program for young children with autism spectrum disorders: a randomized controlled trial. J Dev Behav Pediatr. (2014) 35:475–85. 10.1097/DBP.0000000000000096 25264862 PMC4181375

[71] Wang HI Wright BD Bursnall M Cooper C Kingsley E Le Couteur A Cost-utility analysis of LEGO based therapy for school children and young people with autism spectrum disorder: results from a randomised controlled trial. BMJ Open. (2022) 12:e056347. 10.1136/bmjopen-2021-056347 35039300 PMC8765033

[72] Wright B Kingsley E Cooper C Biggs K Bursnall M Wang HI I-SOCIALISE: results from a cluster randomised controlled trial investigating the social competence and isolation of children with autism taking part in LEGO[(R)] based therapy (‘play brick therapy’) clubs in school environments. Autism. (2023) 27(8):2281–94. 10.1177/13623613231159699 PMC10576908 36991578

[73] Doernberg EA Russ SW Dimitropoulos A. Believing in make-believe: efficacy of a pretend play intervention for school-aged children with high-functioning autism spectrum disorder. J Autism Dev Disord. (2021) 51:576–88. 10.1007/s10803-020-04547-8 32556834

[74] Kasari C Gulsrud A Paparella T Hellemann G Berry K. Randomized comparative efficacy study of parent-mediated interventions for toddlers with autism. J Consult Clin Psychol. (2015) 83:554–63. 10.1037/a0039080 25822242 PMC4755315

[75] Corbett B Blain S Ioannou S Balser M. Changes in anxiety following a randomized control trial of a theatre-based intervention for youth with autism spectrum disorder. Autism. (2017) 21:333–43. 10.1177/1362361316643623 27154909 PMC5633032

[76] Wijnhoven L Creemers DHM Vermulst AA Lindauer RJ Otten R Engels RC Effects of the video game ‘mindlight’ on anxiety of children with an autism spectrum disorder: a randomized controlled trial. J Behav Ther Exp Psychiatry. (2020) 68:101548. 10.1016/j.jbtep.2020.101548 32155470

[77] Vukićević S Đorđević M Glumbić N Bogdanović Z Đurić Jovičić M. A demonstration project for the utility of kinect-based educational games to benefit motor skills of children with ASD. Percept Mot Skills. (2019) 126(6):1117–44. 10.1177/0031512519867521 31390305

[78] Sarah B Parson J Renshaw K Stagnitti K. Can children’s play themes be assessed to inform play therapy practice? Clin Child Psychol Psychiatry. (2021) 26:257–67. 10.1177/1359104520964510 33100021 PMC7802051

[79] Macoun SJ Schneider I Bedir B Sheehan J Sung A. Pilot study of an attention and executive function cognitive intervention in children with autism spectrum disorders. J Autism Dev Disord. (2021) 51:2600–10. 10.1007/s10803-020-04723-w 33029666

[80] Barnett W Hansen CL Bailes LG Humphreys KL. Caregiver-child proximity as a dimension of early experience. Dev Psychopathol. (2022) 34:647–65. 10.1017/s0954579421001644 35074028

[81] Rosales FJ Reznick JS Zeisel SH. Understanding the role of nutrition in the brain and behavioral development of toddlers and preschool children: identifying and addressing methodological barriers. Nutr Neurosci. (2009) 12:190–202. 10.1179/147683009x423454 19761650 PMC2776771

[82] Lebersfeld JB Swanson M Clesi CD O’Kelley SE. Systematic review and meta-analysis of the clinical utility of the ADOS-2 and the ADI-R in diagnosing autism spectrum disorders in children. J Autism Dev Disord. (2021) 51:4101–14. 10.1007/s10803-020-04839-z 33475930

[83] Fletcher-Watson S McConnell F Manola E McConachie H. Interventions based on the theory of mind cognitive model for autism spectrum disorder (ASD). Cochrane Database Syst Rev. (2014) 2014:Cd008785. 10.1002/14651858.CD008785.pub2 24652601 PMC6923148

[84] Balch JW Ray DC. Emotional assets of children with autism spectrum disorder: a single-case therapeutic outcome experiment. J Couns Dev. (2015) 93:429–39. 10.1002/jcad.12041

[85] Arkush L Smith-Collins AP Fiorentini C Skuse DH. Recognition of face and non-face stimuli in autistic spectrum disorder. Autism Res. (2013) 6:550–60. 10.1002/aur.1318 23894016

[86] Corbett BA Swain DM Coke C Simon D Newsom C Houchins-Juarez N Improvement in social deficits in autism spectrum disorders using a theatre-based, peer-mediated intervention. Autism Res. (2014) 7:4–16. 10.1002/aur.1341 24150989 PMC3943749

[87] Stavropoulos KK Carver LJ. Research review: social motivation and oxytocin in autism–implications for joint attention development and intervention. J Child Psychol Psychiatry. (2013) 54:603–18. 10.1111/jcpp.12061 23451765 PMC3663901

[88] Yurkovic-Harding J Lisandrelli G Shaffer RC Dominick KC Pedapati EV Erickson CA Children with ASD establish joint attention during free-flowing toy play without face looks. Curr Biol. (2022) 32:2739–46. e2734. doi: 10.1016/j.cub.2022.04.044 35561679 PMC9233124

[89] Woynaroski T Yoder P Watson LR. Atypical cross-modal profiles and longitudinal associations between vocabulary scores in initially minimally verbal children with ASD. Autism Res. (2016) 9:301–10. 10.1002/aur.1516 26180010 PMC4968579

[90] Ferguson CJ Olson CK. Friends, fun, frustration and fantasy: child motivations for video game play. Motiv Emot. (2013) 37:154–64. 10.1007/s11031-012-9284-7

[91] Granic I Lobel A Engels RCME. The benefits of playing video games. Am Psychol. (2013) 69(1):66. 10.1089/cyber20130296 24295515

[92] Osborne LA McHugh L Saunders J Reed P. Parenting stress reduces the effectiveness of early teaching interventions for autistic spectrum disorders. J Autism Dev Disord. (2008) 38:1092–103. 10.1007/s10803-007-0497-7 18027079

[93] Thomas S White V Ryan N Byrne L. Effectiveness of play therapy in enhancing psychosocial outcomes in children with chronic illness: a systematic review. J Pediatr Nurs. (2022) 63:e72–81. 10.1016/j.pedn.2021.1010.1009 34776315

[94] Wijnhoven L Creemers D Vermulst A Lindauer RJ Otten R Engels RC Effects of the video game ‘mindlight’ on anxiety of children with an autism spectrum disorder: a randomized controlled trial. J Behav Ther Exp Psychiatry. (2020) 68:101548. 10.1016/j.jbtep.2020.101548 32155470

[95] Sameroff A. A unified theory of development: a dialectic integration of nature and nurture. Child Dev. (2010) 81:6–22. 10.1111/j.1467-8624.2009.01378.x 20331651

[96] Bearss K Johnson C Smith T Lecavalier L Swiezy N Aman M Effect of parent training vs parent education on behavioral problems in children with autism spectrum disorder: a randomized clinical trial. Jama. (2015) 313:1524–33. 10.1001/jama.2015.3150 25898050 PMC9078140

[97] Matson JL Matheis M Burns CO Esposito G Venuti P Pisula E Examining cross-cultural differences in autism spectrum disorder: a multinational comparison from Greece, Italy, Japan, Poland, and the United States. Eur Psychiatry. (2017) 42:70–6. 10.1016/j.eurpsy.2016.10.007 28212508

[98] Hus Y. Issues in identification and assessment of children with autism and a proposed resource toolkit for speech-language pathologists. Folia Phoniatr Logop. (2017) 69:27–37. 10.1159/000477398 29248918

